# [Retracted] Long non‑coding RNA receptor activator of nuclear factor‑κB ligand promotes cisplatin resistance in non‑small cell lung cancer cells

**DOI:** 10.3892/etm.2024.12560

**Published:** 2024-05-01

**Authors:** Zhongcheng Zhu, Xiaoyi Gong, Jing Li, Yufeng Shi, Mingyun Zhang

Exp Ther Med 21:518, 2021; DOI: 10.3892/etm.2021.9949

Following the publication of this paper, it was drawn to the Editor's attention by a concerned reader that the ‘Control’ data panel shown for the Transwell migration assay data in Fig. 4E (for the A549 cell line) on p. 6 were strikingly similar to data that had already been published in different form in another article written by different authors at a different research institute [Hua K, Liang C, Miao C, Wang S, Su S, Shao P, Liu B, Bao M, Zhu J, Xu A *et al*: MicroRNA-126 inhibits proliferation and metastasis in prostate cancer via regulation of ADAM9. Oncol Lett 15: 9051-9060, 2018].

Owing to the fact that the contentious data in the above article had already been published prior to its submission to *Experimental and Therapeutic Medicine*, the Editor has decided that this paper should be retracted from the Journal. The authors were asked for an explanation to account for these concerns, but the Editorial Office did not receive a reply. The Editor apologizes to the readership for any inconvenience caused.

